# Rapid Identification of Carbapenemase Genes Directly from Blood Culture Samples

**DOI:** 10.3390/diagnostics15192480

**Published:** 2025-09-28

**Authors:** Ghada A. Ziad, Deena Jalal, Mohamed Hashem, Ahmed A. Sayed, Sally Mahfouz, Ahmed Bayoumi, Maryam Lotfi, Omneya Hassanain, May Tolba, Youssef Madney, Lobna Shalaby, Mervat Elanany

**Affiliations:** 1 Microbiology Unit, Children’s Cancer Hospital Egypt (CCHE-57357), Cairo 11441, Egypt; ghada.aly@57357.org; 2 Genomic and Metagenomics Research Program, Department of Basic Research, Children’s Cancer Hospital Egypt (CCHE-57357), Cairo 11441, Egypt; deena.jalal@57357.org (D.J.); ahmad.sayed@57357.org (A.A.S.); mariam.lotfi@57357.org (M.L.); 3 Research Department, Children’s Cancer Hospital Egypt (CCHE-57357), Cairo 11441, Egypt; mohamed.diaaeldin@57357.org (M.H.); omneya.hassanain@57357.com (O.H.); 4 Department of Biochemistry, Faculty of Science, Ain Shams University, Cairo 11566, Egypt; 5 Pediatric Oncology Department, Children’s Cancer Hospital Egypt (CCHE-57357), Cairo 11441, Egypt; sally.mahfouz@57357.org (S.M.); ahmed.kamal@y7mail.com (A.B.); yousif.madney@57357.org (Y.M.); lobna.shalaby@57357.org (L.S.); 6 Pediatric Oncology Department, National Cancer Institute (NCI), Cairo University, Cairo 11562, Egypt; 7 Department of Pharmaceutical Services and Sciences, Children’s Cancer Hospital Egypt (CCHE-57357), Cairo 11441, Egypt; may.m.hassan.a@gmail.com; 8 Clinical Pathology Department, Faculty of Medicine, Cairo University, Cairo 11562, Egypt

**Keywords:** blood culture, carbapenemase genes, Xpert^®^ Carba-R assay, Whole-Genome Sequencing (WGS), diagnostic microbiology

## Abstract

**Background/Objectives:** The rapid identification of carbapenemase genes directly from positive blood culture (BC) samples shortens the time needed to initiate optimal antimicrobial therapy for Carbapenemase-Producing Enterobacterales (CPE) infections. Several commercial automated PCR systems are available for detecting CPE resistance genes but are expensive. The Xpert^®^ Carba-R assay (Cepheid GeneXpert System) has high sensitivity and specificity for the detection of carbapenamase genes from bacterial colonies or rectal swabs, with an affordable price. This assay was not used for positive BC testing of CPE resistance genes. Whole-Genome Sequencing (WGS) for resistance genes can be used as the gold standard at a research level. In this study, we evaluated the performance of the Xpert^®^ Carba-R assay for the early detection of carbapenamase genes directly from positive BCs, using WGS as the gold standard. **Methods:** A prospective observational study was conducted at Children’s Cancer Hospital-Egypt (CCHE-57357). All positive BCs underwent direct gram staining and conventional cultures. A total of 590 positive BCs containing Gram-negative rods (GNRs) were identified. The Xpert^®^ Carba-R assay was used to detect carbapenemase genes directly from the positive BC bottle compared with WGS results. **Results:** Among the 590 GNR specimens, 178 were found to carry carbapenemase genes using the Xpert^®^ Carba-R assay, with results obtained in approximately one hour. The main genotypes detected were *bla*_NDM_, *bla*_OXA-48_-like, and dual *bla*_NDM_/*bla*_OXA-48_-like at 27%, 29%, and 33%, respectively. The agreement between Xpert^®^ Carba-R assay and WGS results was almost perfect for the genotype resistance pattern of isolates and individual gene detection. **Conclusions:** The use of the Xpert^®^ Carba-R assay directly from BC bottles was an easy-to-use, time-saving, affordable tool with high accuracy in identifying carbapenemase genes and, thus, shortens the time needed to initiate optimal antimicrobial therapy for CPE infections.

## 1. Introduction

Antimicrobial resistance (AMR) is widely recognized as one of the most pressing global health challenges of the 21st century, with profound clinical, economic, and public health consequences. Among healthcare-associated infections, bloodstream infections (BSIs) caused by extended-spectrum beta-lactamase (ESBL)-producing and carbapenemase-producing Enterobacterales (CPE) are associated with substantial morbidity and mortality worldwide. Such infections often extend hospitalization, escalate treatment expenses, and place additional strain on healthcare systems, with mortality rates reported as high as 44% in some patient populations [1]. Enterobacterales include some of the most common Gram-negative pathogens. The World Health Organization (WHO) has declared CPEs as critical priority pathogens for novel drug development [2]. In Egypt, 54% of Enterobacterales healthcare-associated infections (HAIs) are CPE, with high mortality rates and more frequently isolated from blood specimens [3]. Among the CPE, the New Delhi Metallo-beta-lactamase NDM gene is the most prevalent genotype, detected in (68.9%) of CPE cases in Egypt [4].

Traditional phenotypic methods for detecting carbapenem resistance and ESBL production rely on culture-based susceptibility testing, which may require 48 h or longer to yield results. This delay contributes to the administration of inappropriate or delayed antimicrobial therapy that can worsen clinical outcomes. Rapid identification of CPE directly from positive BC allows for early, targeted interventions, including prompt patient isolation and the initiation of contact precautions that help break the transmission chain in healthcare environments. Automated real-time PCR assays, such as the Xpert^®^ Carba-R assay (Cepheid GeneXpert System), enable the direct detection of major carbapenemase genes—including *bla*_NDM_, *bla*_KPC_, *bla*_VIM_, *bla*_OXA-48_-like, and *bla*_IMP_—from positive BCs in about an hour. While the assay evaluated in this study has demonstrated potential for use with positive BCs, it is important to note that, as per the manufacturer’s current specifications, it is approved only for use with cultured isolates and rectal swabs. Its application to BC samples remains investigational for research purposes only. These assays are affordable for broad clinical adoption, showing concordance rates of up to 96% when compared to DNA sequencing as a gold standard [5].

Rapid immunochromatographic lateral flow assays (LFIAs), such as NG-Test CTX-M MULTI and NG-Test Carba 5, have emerged as pragmatic, cost-effective tools that detect CTX-M-type ESBLs and the five main carbapenemases directly from positive BC broths within a short time frame, typically less than an hour. Multicenter studies have demonstrated sensitivities and specificities near 100%, affirming their reliability [6,7].

Only few assays work directly on BCs [8]. The FilmArray^®^ (Biomérieux, Marcy l’Etoile, France) and GenMark’s ePlex panels can detect *bla*_NDM_, *bla_KPC_, bla_VIM_, bla_OXA-48_*-like, and *bla_IMP_* genes, with results obtained in one hour, and provide rapid microbial identification, but they are more expensive than the Xpert^®^ Carba-R assay Test. This acceleration is critical to antimicrobial stewardship, allowing for early and targeted antimicrobial therapy and minimizing unnecessary broad-spectrum antibiotic use [9].

Whole-Genome Sequencing (WGS) is the most comprehensive molecular method to detect AMR, because it allows the simultaneous detection of resistance to numerous antimicrobial classes. The major limitations to the widespread adoption of WGS-based antimicrobial susceptibility testing (AST) in clinical laboratories remain the very high cost, turn-around time, bioinformatics expertise, lack of well-defined validation criteria, and the robust collection of validation materials [10].

CPEs are the leading cause of febrile neutropenia at Children’s Cancer Hospital-Egypt (CCHE-57357), the largest pediatric oncology hospital in Egypt and the Middle East. This makes the implementation of rapid diagnostics essential for optimizing patient management and timely isolation. Few studies have validated the performance of the Xpert^®^ Carba-R assay for the rapid detection of main carbapenemase genes directly from positive BCs [5,8,11]. The current study aimed to evaluate the performance of the Xpert^®^ Carba-R assay for the early detection of CPE resistance genes directly from positive BCs compared with WGS as the reference standard.

## 2. Materials and Methods

### 2.1. Setting and Study Design

**Figure 1:**
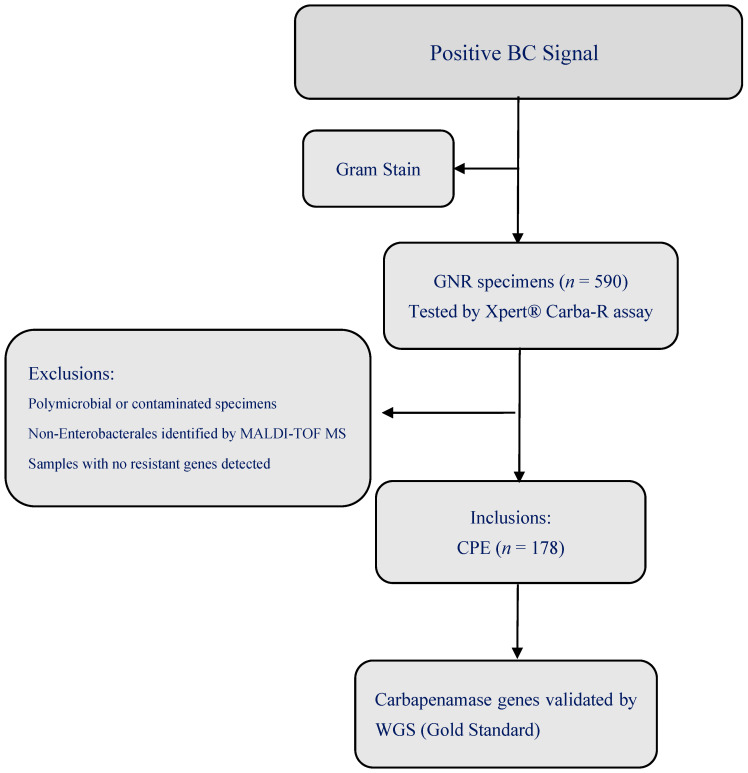
Study flow diagram. The diagram illustrates the diagnostic pathway from initial blood culture positivity to molecular testing using the Xpert^®^ Carba-R assay and subsequent validation by WGS. BC: Blood Culture; GNR: Gram-Negative Rods; CPE: Carbapenemase Producing Enterobacterales; WGS: Whole-Genome Sequencing; MALDI-TOF MS: Matrix-Assisted Laser Desorption/Ionization Time-of-Flight Mass Spectrometry.

A single-center, prospective, observational study conducted at the CCHE-57357. The hospital houses approximately 300 inpatient beds, with approximately 3000 new admissions per year for children with an underlying malignancy (Figure 1).

### 2.2. Study Population

Children and adolescents (≤18 years old) with positive BCs collected from July 2021 to June 2023. Only the first positive monomicrobial BC per patient was included, while positive BCs with polymicrobial pathogens or contaminated specimens were excluded. The study protocol was approved by the Institutional Review Board at the Children’s Cancer Hospital-Egypt.

### 2.3. Microbiological Methods

BCs were monitored using the following automated blood culture continuous monitoring systems: the BD BACTEC™ 9240 blood culture system (Becton Dickinson, Franklin Lakes, NJ, USA) and the BacT/ALERT^®^ 3D System (bioMeriéux, Marcy-l’Étoile, France). All positive BCs were subjected to direct gram staining (30 min), sub-cultured on MacConkey agar and blood agar plates (Oxoid, Hampshire, UK), and incubated at 35–37 °C for 18–24 h according to CLSI standards [12]. Only GNRs were subjected to the Xpert^®^ Carba-R assay.

A total of 590 positive BC samples that were positive for GNRs were identified by gram staining and tested by the Xpert^®^ Carba-R assay directly from BC to GenXpert machine. After short subcultures (3–4 h), the isolates were identified by Matrix-Assisted Laser Desorption/Ionization Time-of-Flight (MALDI TOF) VITEK^®^ MS (VMS, bioMerieux, Marcy-l’Étoile, France), which took 30 min. One hundred and seventy-eight CPE isolates were included while the rest were excluded because they were either non-Enterobacterales or had no resistant genes detected. These CPE isolates were stored at −80 °C in sterile glycerol nutrient broth for further validation by WGS as a gold standard [13]. The results of the Xpert^®^ Carba-R assay were evaluated against the WGS results.

### 2.4. Xpert^®^ Carba-R Assay

The Xpert^®^ Carba-R assay (Cepheid GeneXpert System, Sunnyvale, CA, USA) utilizes real-time PCR to detect the five major carbapenemase gene families, including *bla*_IMP_ (*bla*_IMP-1_*,*_3,6,10,25&30_), All *bla*_KPC_, All *bla*_NDM_, *bla*_OXA-48_-like (*bla*_OXA-48,162,163,181,204,232,244,245&247_), and All *bla*_VIM_.

According to current manufacturer instructions for use, the assay is validated for use with cultured bacterial isolates and rectal swabs, demonstrating a sensitivity and specificity of 96.6% and 98.6%, respectively, for these sample types [14]. In this study, the Xpert^®^ Carba-R assay was applied to positive BCs for research purposes only, to evaluate its potential for rapid carbapenemase gene detection directly from these specimens. The verification of its performance for this specimen type was conducted as part of the present study. In summary, a 50 μL aliquot from the BC was directly mixed with Sample Reagent Buffer and vortexed at a high speed for 10 s. Then, 1.7 mL of this sample elution reagent was transferred to the specimen chamber of an Xpert cartridge, which was detected on the GeneXpert platform. The results were interpreted by the GeneXpert System in 50 min. The cost of the Xpert^®^ Carba-R test is $31 per sample [15].

### 2.5. Whole-Genome Sequencing (WGS): (Figure 2)

The frozen strains were sub-cultured on MacConkey agar media (Oxoid, Hampshire, UK) and incubated at 35–37 °C for 18–24 h in the microbiology laboratory. DNA was extracted using the PureLink Microbiome DNA Extraction Kit (Invitrogen, Carlsbad, CA, USA), and libraries were prepared using the Next era XT DNA Library Preparation Kit (Illumina, San Diego, CA, USA) for sequencing on an Illumina platform. Assemblies were generated using Unicycler (Wick et al., 2017 [16]), and quality control metrics demonstrated a robust sequencing performance, with mean coverage depth ranging from 11.32× to 158.41× (median: 45.24×), contig counts of 87–848 (median: 198), and N50 values from 5662 bp to 467,150 bp (median: 106,193 bp). Total assembled lengths (4.6–6.5 Mbp) and GC content (50.4–57.5%) were consistent with expected Enterobacterales genomic characteristics. Antimicrobial resistance genes were identified from assembled contigs using the NCBI AMRFinderPlus database [16,17]. The WGS process in the original study required approximately three weeks and cost $200 per sample when processed in batches of at least 20 isolates.

### 2.6. Statistical Analysis

Diagnostic parameters were calculated using the Kappa statistic to measure the level of agreement between the Xpert^®^ Carba-R assay and the gold standard, WGS, while accounting for the possibility of agreement occurring by chance. The Kappa value was interpreted according to standard guidelines, where values of 0.61–0.80 represent “Substantial” agreement and 0.81–1.00 represent “Almost Perfect” agreement. A *p*-value was calculated for each Kappa statistic to test the null hypothesis that the observed agreement was no better than chance. This analysis was performed using the irr package in R version 4.5.1. For all statistical tests, a *p*-value of less than 0.05 was considered statistically significant.

## 3. Results

### 3.1. Overview of Clinical Isolates

Xpert^®^ Carba-R testing was performed on 590 BC samples that were positive for GNR on Gram staining. Of these, only 178 clinical isolates (30%) were CPEs. *Klebsiella pneumoniae* and *Escherichia coli* were the most frequently identified species, with other Enterobacterales detected less often (Table 1). The distribution of carbapenemase genes and their combinations are detailed in Table 2 and Supplementary Table S1.

### 3.2. Performance of the Xpert^®^ Carba-R Assay

A total of 178 samples were analyzed to evaluate the diagnostic performance of the Xpert^®^ Carba-R against WGS as the reference standard. Table 3 summarizes the concordance carbapenemase phenotypes, while Table 4 provides full performance metrics. Based on sequencing, *bla*_NDM_ and *bla*_OXA-48_-like were the most prevalent genes, followed by *bla*_KPC_ and *bla*_VIM_; no *bla*_IMP_-positive samples were detected. The assay demonstrated high sensitivity and specificity for all targets, with agreement with WGS ranging from 88.9% to 100%, and κ values from 0.88 to 0.98. Minor discrepancies, including a small number of false-positive results for *bla*_NDM_ and *bla*_OXA-48_-like, are detailed in Table 5.

The low cost (31$/test) and rapid result of the Xpert^®^ Carba-R assay (less than one hour) was noted when compared to the high cost (200$/test) and long testing time (three weeks) of WGS.

## 4. Discussion

The emergence of CPEs presents significant challenges for clinical microbiology, infection control, infectious disease management, and antimicrobial stewardship programs worldwide [18,19,20,21]. The ability to detect carbapenemase genes rapidly and accurately is critical in mitigating the global spread of multidrug-resistant organisms. This study evaluated the Xpert^®^ Carba-R assay, originally developed for rectal swabs and isolated cultures, to determine its effectiveness in directly detecting carbapenemase genes in many positive BCs. The assay enables the identification of major carbapenemase-encoding genes within less than an hour. The Xpert^®^ Carba-R assay is clinically useful not only for the rapid identification of CPE but also for predicting risks of infection [22].

The evaluation focused on the assay’s performance in comparison to the gold standard WGS. The Xpert^®^ Carba-R assay highlighted notable patterns in gene detection. Among these, the co-occurrence of *bla*_NDM_ and *bla*_OXA-48_-like genes was the most prevalent (33%), followed by *bla*_OXA-48_-like (29.8%) and *bla*_NDM_ (27.5%). The study proved that 38.2% of isolates carried more than one resistance gene. These findings align with other studies conducted in Egypt, which reported *bla*_NDM_ and *bla*_OXA-48_-like genes as the predominant carbapenemase-encoding genes in Enterobacterales, with prevalence rates ranging from 26.0% to 68.8% and 30% to 58.6%, respectively, and 32.3% of isolates carried more than one resistance gene [23]. This alignment between studies shows the trend of different CPE genotypes in Egypt.

The Xpert^®^ Carba-R assay demonstrated exceptional performance when applied directly to BC samples, underscoring its potential clinical utility. Agreement with WGS was almost perfect for *bla*_NDM_, *bla*_KPC_, *bla*_VIM_, and *bla*_OXA-48_-like detection. When considering the combined performance across all carbapenemase targets, the assay achieved an overall sensitivity of 99.2%, specificity of 98.9%, PPV of 97.2%, and NPV of 99.7%. Another study that tested the Xpert^®^ Carba-R assay on stored CPE isolates similarly revealed that the overall accuracy of the Xpert^®^ Carba-R assay was 98%, with a sensitivity of 98% and specificity of 97% [24]. Low isolate counts for certain gene targets—particularly *bla*_VIM_, *bla*_KPC_, and *bla*_IMP_—may inflate performance estimates due to wide confidence intervals. This limitation underscores the need for cautious interpretation, especially when extrapolating results to settings with different epidemiological profiles. The observed false negatives (including *bla*_OXA-244_, *bla*_KPC_, and *bla*_NDM_) could be attributed to factors such as low target gene copy numbers near the assay’s limit of detection, sequence variants not optimally recognized by the primers/probes, mixed infections, or prior antimicrobial exposure reducing bacterial load. Conversely, false positives (e.g., *bla*_OXA-48_-like, *bla*_NDM_, and *bla*_VIM_) may result from cross-reactivity with non-carbapenemase variants, sample contamination, or degraded nucleic acids. From a clinical perspective, both false negatives and false positives carry significant implications: missed detections may delay targeted therapy and infection control measures, while false positives can lead to unnecessary isolation, inappropriate antimicrobial use, and increased costs. Integrating rapid molecular results with confirmatory culture-based AST and local epidemiological data is therefore essential to guide optimal patient management and antimicrobial stewardship interventions.

The agreement with WGS was almost perfect for *bla*_NDM_, *bla*_KPC_, *bla*_OXA-48_-like_,_ and *bla*_VIM_. Based on these findings—together with its low cost and rapid turnaround—the antimicrobial stewardship program in our center approved the use of this protocol.

Compared to conventional culture-based methods, which can require 48–72 h, the Xpert^®^ Carba-R assay provides results in under one hour. This is on par with other rapid diagnostics, such as NG-Test Carba 5 and lateral flow immunoassays (LFIAs), which also detect carbapenemase genes in under an hour. However, unlike these methods, the Xpert^®^ Carba-R assay provides precise gene identification (e.g., *bla*_NDM_ and *bla*_KPC_), making it suitable for epidemiological surveillance and informing targeted therapies.

Compared with other rapid diagnostic platforms, such as NG-Test Carba 5, FilmArray^®^, and ePlex, the Xpert^®^ Carba-R assay offers a balance of speed, accuracy, and operational simplicity. Its rapid turnaround can facilitate earlier infection control interventions and targeted antimicrobial therapy, potentially reducing transmission during outbreaks. In resource-limited settings, the assay’s cost-effectiveness and minimal infrastructure requirements make it a feasible option. Looking forward, integrating rapid molecular diagnostics with phenotypic susceptibility testing and antimicrobial stewardship programs could further optimize patient outcomes and curb resistance spread.

Furthermore, MALDI-TOF MS, used for organism identification, complements molecular resistance profiling but does not directly detect resistance genes. The integration of the Xpert^®^ Carba-R assay with MALDI-TOF supports comprehensive workflows for rapid pathogen detection and resistance profiling.

The discordant results with WGS are also discussed. All were in isolates harboring dual genes. Variants of genes may evade detection if their sequences differ significantly from those targeted by the assay. This is especially true in polymicrobial infections or cases involving prior antibiotic treatment [5,22]. Analytical studies have further shown that, in case of the presence of two bacterial populations in the same colony, when one carbapenemase-producing bacterial species is inoculated near the limit of detection (LoD; the low concentration of target that can be reliably detected by the assay) and another one is present at concentrations equal or greater than the LoD, the low concentration target may not be detected [14].

The test failed to detect *bla*_OXA-244_ gene of E. coli harboring *bla*_NDM_*/bla*_OXA-244_, which presents a healthcare problem. One study proved that *bla*_OXA-244_-producing *E. coli* isolates are difficult to detect, which may lead to their silent spread [25]. Other false negatives were one *bla*_KPC_ and one *bla*_NDM_-producing isolate, which may pose a delay in target therapy initiation, but rapid diagnostics must be followed by routine culture and susceptibility tests to confirm the result [26]. Other authors have explained that the false-negative assay’s sensitivity may also be insufficient if carbapenemase-producing bacteria are present in low quantities. Mutations within carbapenemase genes may result in false negatives due to challenges in primer and probe binding [5]. All these factors may explain the false-negative results.

The test showed false-positive *bla*_OXA-48_-like genes in three samples, a false-positive *bla*_NDM_ gene in one sample, and one *bla*_VIM_ false-positive result. This may result in unnecessary treatment and patient isolation with increased cost [27]. Moreover, the quality of clinical samples plays a critical role in detection efficacy. Factors such as sample contamination, improper handling, and nucleic acid degradation can significantly affect the test and may result in a false positive. Because of their close relationship with *bla*_OXA-48_-like, three non-carbapenemase variants (*bla*_OXA-163_, *bla*_OXA-247_, and *bla*_OXA-405_) usually give false-positive results with molecular methods. The primers used in this assay are designed to amplify a broad range of *bla*_OXA-48_-like sequences, hence can lead to the detection of variants that may not be clinically relevant in terms of carbapenem resistance. In many studies, most discrepant results were non-KPC genes, like our center’s results [28,29,30].

Globally, the prevalence of *bla*_IMP_-producing carbapenem-resistant Enterobacterales is highest in Asia and Oceania, while these strains remain uncommon in Egypt [5,23]. Additionally, many rapid commercially available assays for carbapenemase detection, such as PCR, either fail to identify *bla*_IMP_ genes altogether or are limited to specific variants, such as *bla*_IMP-1_, which may not represent the most prevalent types [31]. In this study, *bla*_IMP_ genes were negative by both WGS and Xpert^®^ Carba-R assay, confirming the absence or rarity of this gene in the Middle East. Few studies have validated the performance of the Xpert^®^ Carba-R assay for the rapid detection of the main carbapenemase genes of Enterobacterales directly from positive BCs, and no study has compared the performance of the Xpert^®^ Carba-R assay to WGS for Enterobacterales from BCs. So, we consider this as innovative research. However, by this approach, the detection of carbapenemase genes does not always indicate phenotypic expression, and conventional cultures and AST must be conducted for every sample.

## 5. Limitations

This study has several limitations. First, the number of isolates carrying certain carbapenemase genes (e.g., *bla*_VIM_, *bla*_KPC_, and *bla*_IMP_) was low, which may have inflated performance estimates and widened confidence intervals for these targets. Second, cost-effectiveness metrics linked to individual patient outcomes were not assessed, limiting our ability to evaluate the broader economic impact of implementing this assay. Third, while the Xpert^®^ Carba-R assay provides rapid molecular detection, the presence of a carbapenemase gene does not always equate to phenotypic resistance; therefore, confirmatory culture and antimicrobial susceptibility testing remain essential. Future multicenter studies with larger, more diverse isolate collections and integrated cost-benefit analyses are warranted to validate and extend these results.

## 6. Conclusions

This is the first study to evaluate the performance of the Xpert^®^ Carba-R assay directly from positive BCs against WGS in Enterobacterales. The assay’s rapid turnaround, high accuracy, and affordability support its integration into stewardship and infection control programs, particularly in resource-limited settings. Future multicenter studies should assess its long-term clinical and economic impacts.

## Figures and Tables

**Figure 2:**
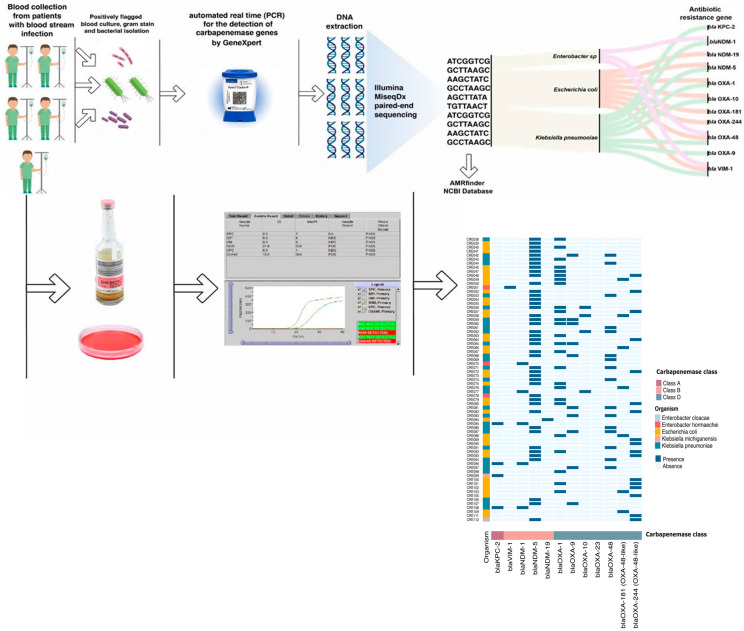
Illustration of laboratory workflow (top: blood culture sample was loaded to an Xpert^®^ Carba-R machine and colonies were subjected to WGS) and (bottom: an example of an Xpert^®^ Carba-R assay result on the machine screen and different genes for different isolates after sequencing).

**Table 1:** Distribution of identified pathogens (*n* = 178 colonies).

Pathogen	Number of Isolates	Percentage (%)
*Klebsiella pneumoniae*	72	40.4
*Escherichia coli*	97	54.5
*Enterobacter* spp.	7	3.9
*Citrobacter* spp.	1	0.6
*Serratia marcescens*	1	0.6
Total	178	100.0

**Table 2:** Distribution of resistance genes identified by Whole-Genome Sequencing (WGS).

Resistance Gene(s)	Number of Isolates	Percentage (%)
*bla_NDM_ & bla_OXA-48_*-like	59	33.1
*bla_NDM_*	49	27.5
*bla_OXA-48_*-like	53	29.8
*bla_KPC-2_ & bla_NDM-1_*	8	4.5
*bla_VIM_*	7	3.9
*bla_KPC-2_*	1	0.6
*bla_VIM_ & bla_OXA-48_*	1	0.6
*bla_IMP_*	–	–
Total	178	100.0

**Table 3:** Concordance between Xpert^®^ Carba-R assay and WGS results for carbapenemase genes (% concordant: percentage of isolates for which the genotype detected by Xpert^®^ Carba-R assay matched the WGS result.).

Bacterial Species	Genotype by WGS		No. of Isolates	No. (% Concordant on Xpert^®^ Carba-R Assay)
*Klebsiella pneumoniae*	*bla*_KPC_	*bla*_KPC-2_	1	1 (100)
	*bla*_NDM_	*bla*_NDM-1_	3	3 (100)
		*bla*_NDM-5_	10	10 (100)
		*bla*_NDM-9_	2	2 (100)
	*bla*_OXA-48_-like	*bla*_OXA-48_	10	10 (100)
		*bla*_OXA-181_	5	5 (100)
	*bla*_IMP_	*bla*_IMP_	0	0 (100)
	*bla*_VIM_	*bla*_VIM-1_	1	1 (100)
	*bla*_KPC_ */bla*_NDM_	*bla*_KPC-2_/*bla*_NDM-1_	8	7 (87.5)
	*bla*_NDM_/*bla*_OXA-48_	*bla*_NDM-1_/*bla*_OXA-48_	2	2 (100)
		*bla*_NDM-5_/*bla*_OXA-48_	29	29 (100)
*Escherichia coli*	*bla*_KPC_	*bla*_KPC_	0	0 (100)
	*bla*_NDM_	*bla*_NDM-5_	29	27 (93.1)
		*bla*_NDM-19_	2	2 (100)
	*bla*_OXA-48_-like	*bla*_OXA-244_	23	22 (95.6)
		*bla*_OXA-181_/*bla*_OXA-1_	2	2 (100)
		*bla*_OXA-181_	6	6 (100)
		*bla*_OXA-244_/*bla*_OXA-1_	4	4 (100)
		*bla*_OXA-484_	3	2 (66.6)
	*bla*_NDM_/*bla*_OXA-48_-like	*bla*_NDM-5_/*bla*_OXA-48_	2	2 (100)
		*bla*_NDM-5_/*bla*_OXA-244_	21	20 (95.2)
		*bla*_NDM-5_/*bla*_OXA-181_	5	5 (100)
*Enterobacter* spp.	*bla*_VIM_	*bla*_VIM-1_	4	4 (100)
	*bla*_NDM_	*bla*_NDM-1_	1	1 (100)
		*bla*_NDM-5_	1	1 (100)
	*bla*_VIM_/*bla*_OXA-48_-like	*bla*_VIM-1_/*bla*_OXA-48_	1	1 (100)
	*bla*_VIM_*/bla*_OXA-48_/*bla*_NDM_	*bla*_VIM-1_/*bla*_OXA-48_/*bla*_NDM_	0	1 (0)
*Citrobacter* spp.	*bla*_VIM_	*bla*_VIM-1_	1	1 (100)
*Serratia marcescens*	*bla*_VIM_	*bla*_VIM-4_	1	1 (100)

**Table 4:** Diagnostic performance and agreement of Xpert^®^ Carba-R assay compared to WGS.

Gene Target	Sensitivity % (95% CI)	Specificity % (95% CI)	PPV % (95% CI)	NPV % (95% CI)	Cohen’s Kappa (κ)	*p*-Value
*bla*_NDM_	100.0 (97.2–100.0)	96.8 (92.4–100)	98.3 (96.0–100)	100.0 (95.0–100.0)	0.98	<0.0001
*bla*_KPC_	88.9 (68.4–100.0)	100 (98.2–100)	100 (62.6–100)	99.4 (98.3–100.0)	0.94	<0.0001
*bla*_OXA-48_	99.1 (97.4–100.0)	95.4 (90.3–100)	97.4 (94.5–100)	98.4 (95.4–100.0)	0.95	<0.0001
*bla*_VIM_	100.0 (62.6–100.0)	98.8 (97.2–100)	80.0 (55.2–100)	100.0 (98.2–100.0)	0.99	<0.0001
*bla*_IMP_	N/A *	100.0 (98.3–100.0)	N/A *	100.0 (98.3–100.0)	N/A *	
Overall	99.2 (98.1–100)	98.9 (98.1–99.7)	97.2 (95.2–99.2)	99.7 (99.3–100)	0.98	<0.0001

CI: Confidence Interval; PPV: Positive Predictive Value; NPV: Negative Predictive Value; N/A: Not applicable. * Metrics could not be calculated due to the absence of positive samples in the reference standard cohort.

**Table 5:** Non-concordance between WGS and the Xpert^®^ Carba-R assay.

Bacterial Species	Xpert^®^ Carba-R Assay	WGS
*Klebsiella pneumoniae*	*bla*_NDM_	*bla*_NDM-1_/*bla*_KPC-2_
	*bla*_NDM_/*bla*_OXA-48_-like/*bla*_VIM_	*bla*_NDM-5_/*bla*_OXA-48_
	*bla*_NDM_/*bla*_OXA-48_-like	*bla*_NDM-9_
*Escherichia coli*	*Bla*_NDM_/_OXA-48_-like	*bla*_NDM-5_
	*bla*_NDM_/*bla*_OXA-48_-like	*bla*_NDM-5_
	*bla*_NDM_	*bla*_NDM-5_/*bla*_OXA-244_
	*bla*_NDM_/*bla*_OXA-48_-like	*bla*_OXA-244_
*Enterobacter* sp.	*bla*_OXA-48_-like/*bla*_VIM_	*bla*_NDM-5_/*bla*_OXA-48_/*bla*_VIM-1_

## Data Availability

The datasets generated during and/or analyzed during the current study are available from the corresponding author on reasonable request. WGS data referenced in this study are publicly available under BioProject ID PRJNA1242051 at the NCBI Sequence Read Archive (SRA).

## Supplementary Materials

The following supporting information can be downloaded at: https://www.mdpi.com/article/10.3390/diagnostics15192480/s1, Table S1: Master Excel sheet of gene detections by WGS & Xpert^®^ Carba-R assay.
